# Data for validation and adjustment of APACHE II score in cardiogenic shock patients treated with a percutaneous left ventricular assist device

**DOI:** 10.1016/j.dib.2022.108199

**Published:** 2022-04-22

**Authors:** Johannes Mierke, Thomas Nowack, Tobias Loehn, Franziska Kluge, Frederike Poege, Felix Woitek, Norman Mangner, Karim Ibrahim, Axel Linke, Christian Pfluecke

**Affiliations:** a Technische Universität Dresden, Department of Internal Medicine and Cardiology, Herzzentrum Dresden, University Clinic, Dresden, Germany; b Kreiskrankenhaus Freiberg, Klinik für Innere Medizin II, Freiberg, Germany; c Klinikum Chemnitz, Klinik für Innere Medizin I, Chemnitz, Germany

**Keywords:** Acute Physiology and Chronic Health Evaluation II score, Predicted mortality, Percutaneous left ventricular assist device, Impella CP®, Mechanical circulatory support*.*

## Abstract

A precise prognosis is of imminent importance in intensive care medicine. This article provides data showing the overestimation of intrahospital mortality by APACHE II score in various subgroups of cardiogenic shock patients treated with a percutaneous left ventricular assist device. The data set includes additional baseline characteristics regarding age, pre-existing diseases, characteristics of coronary artery disease, characteristics of cardiopulmonary resuscitation, and hemodynamic parameter not included in the APACHE II score. Further data were provided which characterize derivation and validation group. Both groups were used for adjustment of the APACHE II approach. The data are supplemental to our original research article titled “Predictive value of the APACHE II score in cardiogenic shock patients treated with a percutaneous left ventricular assist device” (Mierke et al., IJC Heart & Vasculature. 40 (2022) 101013. https://doi.org/10.1016/j.ijcha.2022.101013).

## Specifications Table

**Table:** 

Subject	Health and medical sciences
Specific subject area	Cardiology and Cardiovascular Medicine
Type of data	Table
How the data were acquired	Data were collected from the prospective Dresden Impella Registry
Data format	Raw Analyzed
Description of data collection	Data from 180 cardiogenic shock patients (>18 years old), who were included in the unselective Dresden Impella Registry and received left ventricular unloading with Impella CP®, were analyzed. Predicted intrahospital mortality was estimated by APACHE II Score and compared with Kaplan-Meier estimator at survivors’ mean hospital stay lengths ($\hat{S\;}\left(t_{hosp}\right)$).
Data source location	Institution: Technische Universität Dresden, Heart Center Dresden, University Hospital City: Dresden Country: Germany
Data accessibility	Repository name: Mendeley Data DOI: 10.17632/9ktj6nhmbx.1 URL: https://data.mendeley.com/datasets/9ktj6nhmbx/1
Related research article	J. Mierke, T. Nowack, T. Loehn, F. Kluge, F. Poege, U. Speiser, F. Woitek, N. Mangner, K. Ibrahim, A. Linke, C. Pfluecke, Predictive value of the APACHE II score in cardiogenic shock patients treated with a percutaneous left ventricular assist device, IJC Heart & Vasculature. 40 (2022) 101013. https://doi.org/10.1016/j.ijcha.2022.101013.

## Value of the Data

• The database offers baseline characteristics, different clinical parameters and outcome data of cardiogenic shock patients receiving left ventricular unloading with a micro-axial left ventricular assist device (pLVAD).
• The database is useful for exact prediction of outcome in different subgroups of cardiogenic shock patients treated with pLVAD.
• The dataset enables the validation of the adjusted Acute Physiology and Chronic Health Evaluation (APACHE) II score in other cohorts of cardiogenic shock patients treated with pLVAD.
• Researchers with interest in pLVAD in cardiogenic shock can utilize database, combine it with others’ datasets, and analyze them for further insights.
• The dataset can be used for comparison with other cardiogenic shock cohorts treated with another pLVAD than the Impella CP®.

## 1 Data Description

We present data of 180 patients of Dresden Impella Registry with severe CS, who received left ventricular (LV) unloading with a microaxial percutaneous left ventricular assist device (pLVAD). We compared real-world mortality with the predicted mortality estimated by the Acute Physiology and Chronic Health Evaluation (APACHE) II score [1].

Table 1 shows the baseline characteristics of the patients displaying a typical distribution of cardiovascular risk factors found in developed countries. Acute myocardial infarction was the prevailing cause of cardiogenic shock and around 50% of patients received cardiopulmonary resuscitation.

**Table 1:** Baseline characteristics and cause of cardiogenic shock.

Mean ± SEM; (n)		CPR Mean ± SEM; (n)	
Age / a	66.8 ± 1.0; (180)	No / % (n)	50.6 (91)
Male sex / % (n)	70.0 (126)	In-hospital / % (n)	29.4 (53)
BMI / kg/m²	27.3 ± 0.4; (180)	Out-of-hospital / % (n)	20.0 (36)
Diabetes mellitus type II / % (n)	34.7 (61)	Duration CPR / min	28.3 ± 3.1; (70)
		
Hypertension / % (n)	62.5 (110)	**Cause of CS**	
		
Dyslipidaemia / % (n)	42.9 (75)	AMI / % (n)	66.7 (120)
Renal failure / % (n) [eGFR ≤ 60 ml/min/1.73 m²]	21.3 (37)	Decompensated ICM / % (n)	7.8 (14)
		Decompensated Non- ICM / % (n)	11.1 (20)
Atrial fibrillation / % (n)	26.1 (46)	Valvular disease / % (n)	5.0 (9)
PAD / % (n)	6.8 (12)	Interventional complication / % (n)	3.9 (7)
History of stroke / % (n)	8.0 (14)	Heart rhythm disturbances / % (n)	2.2 (4)
History of AMI / % (n)	17.6 (31)	Post cardiac surgery / % (n)	1.1 (2)
History of PCI / % (n)	25.0 (44)	Takotsubo-CMP % (n)	1.1 (2)
History of CABG / % (n)	4.0 (7)	Other1) / % (n)	1.1 (2)

1) Aortic dissection type A BMI, body mass index; PAD, peripheral arterial disease; AMI, acute myocardial infarction; PCI, percutaneous coronary intervention; CABG, coronary artery bypass graft; CPR, cardiopulmonary resuscitation; ICM, ischemic cardiomyopathy; CMP, cardiomyopathy.

Table 2 presents the characteristics of coronary artery disease of the patients with cardiogenic shock complicating acute myocardial infarction (n=120). Coronary three-vessel disease was prevailing in these patients, whereby most frequently culprit lesions of the left coronary system caused an acute myocardial infarction. Percutaneous coronary intervention was the predominant treatment.

**Table 2:** Characteristics of CAD of patients with cardiogenic shock complicating acute myocardial infarction.

(% (n))		
Kind of ACS	NSTEMI	33.3 (40)
	STEMI	66.7 (80)

Culprit lesion	LMS	30.8 (37)
	LAD	40.0 (48)
	RCX	12.5 (15)
	RCA	16.7 (20)

CAD type	1-vessel	17.5 (21)
	2-vessel	30.0 (36)
	3-vessel	52.5 (63)

Treatment	PCI	95.0 (114)
	CABG	1.7 (2)
	Failed PCI	3.3 (4)

Number of treated lesions	1	31.6 (36)
	2	33.3 (38)
	3	24.6 (28)
	≥4	10.5 (12)

Maximum of creatine kinase / µcat/l mean±SEM; (n)		83.1 ± 12.4; (102)

CAD, coronary artery diseases; ACS, acute coronary syndrome; NSTEMI, non-ST-segment elevation myocardial infarction; STEMI, ST-segment elevation myocardial infarction; LMS, left main stem; LAD, left anterior descending; RCX, ramus circumflexus; RCA, right coronary artery; PCI, percutaneous coronary intervention; CABG, coronary artery bypass graft.

Table 3 shows clinical parameters, that are not included in the APACHE II score but are known to influence outcome**.** Patients of the Dresden Impella Registry had high concentration of serum lactate and required intensive ionotropic and vasopressor support in the first 24 hours. The left ventricular ejection fraction was severely impaired before pLVAD.

**Table 3:** Clinical parameters not included in the APACHE II score.

Mean ± SEM; (n)	
Highest serum lactate in first 24 h / mM	9.1 ± 0.4; (180)
Use of NE / % (n)	91.8; (156)
Highest NE dosage in first 24 h / µg/kg/min	0.83 ± 0.08; (151)
Use of dobutamine / % (n)	51.1; (92)
Highest dobutamine dosage in first 24 h / µg/kg/min	7.8 ± 0.6; (92)
LVEF before pLVAD / %	26.2 ± 1.1; (146)
Mechanical ventilation / % (n)	85.4 (140)
Time on mechanical ventilation / h	194.1 ± 22.1; (140)
Length of hospital stay / d	13.8 ± 1.0; (180)
Length of ICU stay / d	11.7 ± 0.9; (180)
Survivors’ length of ICU stay / d	19.2 ± 1.6; (71)
Duration of LV assist / h	53.5 ± 5.3; (179)

APACHE, Acute Physiology and Chronic Health Evaluation; NE, norepinephrine; LVEF, left ventricular ejection fraction; pLVAD, percutaneous left ventricular assist devices; ICU, intensive care unit.

Table 4 compares APACHE II Score, length of hospital stays of survivors, observed mortality, and predicted mortality in different subgroups. The APACHE II score overestimated intrahospital mortality in nearly all sub-categories. The comparisons within the dichotomous or trichotomous subgroups showed no significant difference in observed mortality. The last column of the table displays the adjusted Diagnostic Category Weight, a specific constant for calculation of predicted mortality by the APACHE II score. The approach is described in detail by Mierke et al. [2].

**Table 4:** Comparison of observed and predicted mortality in different subgroups.

Parameter	Sub-category	APACHE II score mean ± SEM; (n)	Survivors’ length of hospital stay [t_hosp_]/ d mean ± SEM; (n)	Mortality at survivors’ mean hospital stay/% $\hat{S\;}\left(t_{hosp}\right)$ ± SE($t_{hosp})$; (n)		Predicted Mortality by APACHE II score / %mean ± SEM; (n)	p-Wert	Adjusted Diagnostic Category Weight
**Sex**	Male	34.0 ± 0.7; (126)	24.1 ± 1.9; (47)	56.3 ± 4.4; (126)	0.480	75.9 ± 2.0; (126)	**<0.001**	-1,194
	Female	32.2 ± 1.0; (54)	20.5 ± 2.7; (24)	50.0 ± 6.8; (54)		71.5 ± 3.0; (54)	**0.018**	-1,184

**CPR**	IHCA	37.4 ± 0.8; (53)	21.5 ± 2.9; (15)	62.3 ± 6.7; (53)	0.660	88.5 ± 1.6; (53)	**0.002**	-1,441
	OHCA	35.8 ± 1.1; (36)	21.7 ± 4.7; (12)	63.9 ± 8.0; (36)		86.0 ± 2.3; (36)	**0.029**	-1,139

**Age**	≤ 50 a	32.0 ± 1.6; (21)	21.4 ± 4.8; (11)	47.6 ± 10.9; (21)	0.938	72.2 ± 5.1; (21)	0.116	/
	> 50 a	33.7 ± 0.6; (159)	23.2 ± 1.7; (60)	54.7 ± 3.9; (159)		74.9 ± 1.7; (159)	**<0.001**	-1,215

**CAD**	No CAD	33.0 ± 0.9; (83)	23.4 ± 2.4; (33)	54.2 ± 5.5; (83)	0.762	72.9 ± 2.7; (83)	**0.010**	-1,133
	CAD	34.1 ± 0.7; (93)	23.3 ± 2.1; (35)	55.9 ± 5.1; (93)		76.5 ± 2.1; (93)	**0.003**	-1,224

**Only acute myocardial infarction patients (n=120)**								

**Sex**	Male	34.7 ± 0.8; (87)	21.6 ± 2.3; (30)	57.5 ± 5.3; (87)	0.247	78.6 ± 2.2; (87)	**0.003**	-1,247
	Female	31.6 ± 1.3; (33)	23.6 ± 3.3; (17)	45.5 ± 8.7; (33)		70.1 ± 3.9; (33)	**0.046**	-1,277

**CPR**	IHCA	36.9 ± 1.1; (38)	21.2 ± 3.6; (11)	60.5 ± 7.9; (38)	0.671	87.3 ± 2.1; (38)	**0.009**	-1,444
	OHCA	36.2 ± 1.2; (29)	23.2 ± 5.2; (10)	62.1 ± 9.0; (29)		86.3 ± 2.8; (29)	**0.036**	-1,274

**Age**	≤ 50 a	32.7 ± 2.5; (10)	13.8 ± 3.9; (5)	50.0 ± 15.8; (10)	0.929	75.1 ± 6.8; (10)	0.350	/
	> 50 a	34.0 ± 0.7; (110)	23.4 ± 2.0; (42)	54.5 ± 4.7; (110)		76.4 ± 2.0; (110)	**<0.001**	-1,267

**Kind of ACS**	STEMI	33.6 ± 0.9; (80)	21.8 ± 2.3; (29)	56.2 ± 5.5; (80)	0.738	75.7 ± 2.5; (80)	**0.007**	-1,139
	NSTEMI	34.3 ± 1.1; (40)	23.2 ± 3.3; (18)	50.0 ± 7.9; (40)		77.4 ± 3.0; (40)	**0.011**	-1,491

**CAD type**	1-CAD	35.1 ± 1.4; (21)	22.7 ± 6.0; (6)	66.7 ± 10.3; (21)	0.073	81.8 ± 3.8; (21)	0.484	/
	2-CAD	33.5 ± 1.6; (36)	20.6 ± 2.7; (18)	41.7 ± 8.2; (36)		73.4 ± 4.2; (36)	**0.009**	-1,709
	3-CAD	33.6 ± 0.9; (63)	23.7 ± 2.9; (23)	57.1 ± 6.2; (63)		76.0 ± 2.5; (63)	**0.023**	-1,103

**Culprit lesion**	LMS	33.8 ± 1.4; (37)	20.4 ± 3.4; (18)	43.2 ± 8.2; (37)	0.181	75.2 ± 3.8; (37)	**0.004**	-1,691
	LAD	33.5 ± 1.0; (48)	21.4 ± 3.2; (15)	62.5 ± 7.0; (48)		76.5 ± 2.9; (48)	0.120	/
	RCX	36.0 ± 1.8; (15)	29.8 ± 5.5; (5)	66.7 ± 12.2; (15)		80.3 ± 5.0; (15)	0.682	/
	RCA	33.0 ± 1.7; (20)	23.6 ± 4.0; (9)	40.0 ± 11.0; (20)		74.6 ± 4.9; (20)	**0.025**	-1,706

CPR, cardiopulmonary resuscitation; IHCA, In-hospital cardiac arrest; OHCA, Out-of-hospital cardiac arrest; CAD, coronary artery diseases; ACS, acute coronary syndrome; STEMI, ST-segment elevation myocardial infarction; NSTEMI, non-ST-segment elevation myocardial infarction; LMS, left main stem; LAD, left anterior descending; RCX, ramus circumflexus; RCA, right coronary artery; PCI, percutaneous coronary intervention; CABG, coronary artery bypass graft.

Table 5 presents the baseline characteristics of derivation and validation group, which were obtained by random division o thef total study cohort. These groups were used for adjustment of Diagnostic Category Weight and its internal validation. The derivation and validation group showed well balanced baseline characteristics. Differences were only observed between body mass index and occurrence of peripheral arterial disease.

**Table 5:** Baseline characteristics of validation and derivation cohort.

Mean ± SEM; (n)				CPR Mean ± SEM; (n)			
	Validation group	Derivation group	p-value		Validation group	Derivation group	p-value
Age / a	68.1 ± 1.3; (90)	65.5 ± 1.4; (90)	0.261	No / % (n)	48.9 (44)	52.2 (47)	0.655
Male sex / % (n)	66.7 (60)	73.3 (66)	0.329	In-hospital / % (n)	30.0 (27)	28.9 (26)	0.870
BMI / kg/m²	26.5 ± 0.4; (90)	28.2 ± 0.6; (90)	**0.043**	Out-of-hospital / % (n)	21.1 (19)	18.9 (17)	0.709
BSA / m²	1.92 ± 0.02; (90)	2.00 ± 0.02; (90)	0.058	Duration CPR / min	26.9 ± 4.5; (36)	29.7 ± 4.2; (34)	0.306
				
Diabetes mellitus type II / % (n)	36.4 (32)	33.0 (29)	0.635	**Cause of CS**			
				
Hypertension / % (n)	63.6 (56)	61.4 (54)	0.755	AMI / % (n)	68.9 (62)	64.4 (58)	0.527
Dyslipidaemia / % (n)	37.5 (33)	48.3 (42)	0.150	Decompensated ICM / % (n)	7.8 (7)	7.8 (7)	1.000
Renal failure / % (n) [eGFR ≤ 60 ml/min/1.73 m²]	24.4 (21)	18.2 (16)	0.315	Decompensated Non- ICM / % (n)	11.1 (10)	11.1 (10)	1.000
Atrial fibrillation / % (n)	26.1 (23)	26.1 (23)	1.000	Valvular disease / % (n)	5.6 (5)	4.4 (4)	1.000
PAD / % (n)	11.4 (10)	2.3 (2)	**0.032**	Interventional complication / % (n)	1.1 (1)	6.7 (6)	0.118
History of stroke / % (n)	11.4 (10)	4.5 (4)	0.162	Heart rhythm disturbances / % (n)	2.2 (2)	2.2 (2)	1.000
History of AMI / % (n)	14.8 (13)	20.5 (18)	0.322	Post cardiac surgery / % (n)	1.1 (1)	1.1 (1)	1.000
History of PCI / % (n)	28.4 (25)	21.6 (19)	0.296	Takotsubo-CMP % (n)	1.1 (1)	1.1 (1)	1.000
History of CABG / % (n)	3.4 (3)	4.5 (4)	1.000	Other1) / % (n)	1.1 (1)	1.1 (1)	1.000

1) Aortic dissection type A; BMI, body mass index; BSA, body surface area; PAD, peripheral arterial disease; AMI, acute myocardial infarction; PCI, percutaneous coronary intervention; CABG, coronary artery bypass graft; CPR, cardiopulmonary resuscitation; ICM, ischemic cardiomyopathy; CMP, cardiomyopathy.

Individual raw data on outcome and on all measured parameters are listed in a supplementary Excel sheet.

## 2 Experimental Design, Materials and Methods

Clinical data were collected from the prospective Dresden Impella Registry during the period from February 2014 to May 2018. The predicted intrahospital mortality estimated by APACHE II score was calculated as described by Knaus et al. [1] and compared with the registry mortality. The comparison was performed conservatively by using Kaplan-Meier estimator at survivors’ length of hospital stay. Patients who died intrahospital were excluded from the calculation of length of hospital stay. Receiver operating characteristics (ROC) analysis was performed to prove the accuracy of APACHE II score. In case of an overestimation of predicted mortality and an acceptable accuracy of APACHE II score, a specific constant (Diagnostic Category Weight) for calculation of predicted mortality was adjusted. For this purpose, the total study cohort was randomly divided into a derivation and a validation group. The derivation group was used for the calculation of the adjusted Diagnostic Category Weight (DCW). The observed mortality of the validation group was compared with predicted mortality calculated by adjusted Diagnostic Category Weight by using two approaches. First, goodness of fit was proved by the Hosmer-Lemeshow statistics. Second, differences between observed and predicted mortality by using either original DCW or adjusted DCW were compared. Finally, the adjusted DCW was calculated for every subgroup which showed a significant difference between observed and predicted mortality.

## Ethics Statement

The study was performed in accordance with the Helsinki Declaration and local law and was approved by the institutional ethics committee of the Technische Universität Dresden (EK 457-122-014). All patients were adequately informed about the objective of the study and presented data are anonymized.

## CRediT Author Statement

**Johannes Mierke:** Conceptualization, Methodology, Software, Formal analysis, Writing – original draft; **Thomas Nowack:** Methodology, Software, Formal analysis; **Tobias Loehn:** Conceptualization, Methodology; **Franziska Kluge:** Investigation; **Frederike Poege:** Investigation; **Felix Woitek:** Data Curation; **Norman Mangner:** Data curation; **Karim Ibrahim:** Writing – review & editing; **Axel Linke:** Supervision, Writing – review & editing; **Christian Pfluecke:** Writing – review & editing.

## Declaration of Competing Interest

The authors declare that they have no known competing financial interests or personal relationships that could have appeared to influence the work reported in this paper.

The authors declare the following financial interests/ personal relationships which may be considered as potential competing interests:

**FW** reports personal fees from Abiomed, Abbott, Biotronik, Boston Scientific, Corvia, MSD, NeoVasc, outside the submitted work.

**NM** reports personal fees from Abiomed, Edwards LifeScience, Medtronic, Biotronik, Novartis, Sanofi Genzyme, Bayer, Pfizer, and AstraZeneca, outside the submitted work.

**AL** reports grants from Novartis, personal fees from Medtronic, Abbott, Edwards Lifesciences, Boston Scientific, Astra Zeneca, Novartis, Pfizer, Abiomed, Bayer, Boehringer, and other from Picardia, Transverse Medical, Claret Medical, outside the submitted work.

The other authors have disclosed that they do not have any potential conflicts of interest.

## Data Availability

Data for validation and adjustment of APACHE II score in cardiogenic shock patients treated with a percutaneous left ventricular assist device (Original data) (Mendeley Data).
